# Efficacy of Ultrasound-Guided Erector Spinae Plane Block in Percutaneous Nephrolithotomy

**DOI:** 10.7759/cureus.40186

**Published:** 2023-06-09

**Authors:** Satya P Pandey, Urvashi Yadav, Mohd Mubashir A Khan, Amit K Singh, Shipra Verma, Shuchi Nigam

**Affiliations:** 1 Anaesthesiology, Uttar Pradesh University of Medical Sciences, Etawah, IND; 2 Urosurgery, Uttar Pradesh University of Medical Sciences, Etawah, IND; 3 Anaesthesiology, Shaikh-Ul-Hind Maulana Mahmood Hasan Medical College, Saharanpur, IND

**Keywords:** tramadol, postoperative pain, nerve block, local anesthetics, interventional ultrasonography, analgesia

## Abstract

Background

Percutaneous nephrolithotomy (PCNL) is presently the preferred method for managing renal calculi. Visceral pain from the kidney and ureter and somatic pain from the incision site are the primary causes of immediate postoperative pain following PCNL. Poor pain control is associated with unwanted consequences such as patient discomfort, delayed recovery, and prolonged hospital stay. Recently, the erector spinae plane (ESP) block has been used in many thoracic and abdominal surgeries for the control of postoperative pain. In this study, we aimed to assess the effectiveness of the ultrasound-guided ESP block following PCNL.

Methodology

This was a prospective, double-blind, randomized controlled study including 60 patients who were scheduled for elective PCNL under general anesthesia. Patients were randomly divided into two groups. Group E underwent an ultrasound-guided ESP block with 20 mL of the local anesthetic mixture at the T-9 level unilaterally on the side of surgery, and group C was a sham group in which 20 mL of normal saline was injected on the side of surgery. Changes in postoperative pain score were the primary outcome, and the duration of analgesia, the total analgesic requirement in 24 hours, and patient satisfaction were the secondary outcomes.

Results

The demographic data of both groups were comparable. The Visual Analog Scale score was considerably lower in group E than in group C at two, four, six, and eight hours postoperatively. In group E, the mean analgesic duration was substantially longer than that in group C (8.87 ± 2.45 hours vs. 5.67 ± 1.58 hours, respectively). The tramadol requirement was higher in group C (286.67 ± 62.88 mg) than in group E (133.33 ± 47.95 mg) during the 24-hour postoperative period. At 12 hours, patient satisfaction was considerably higher in group E than in group C (6.73 ± 0.45 vs. 5.87 ± 0.35, respectively).

Conclusions

The ultrasound-guided ESP block provided efficient postoperative pain relief, prolonged duration of analgesia, and reduced tramadol intake after PCNL surgery.

## Introduction

Percutaneous nephrolithotomy (PCNL) is presently the preferred method for the management of renal calculi. PCNL benefits from avoiding the extensive incision of open surgery, leading to early ambulation, shorter hospital stays, and lower morbidity. Visceral pain from the kidney and ureter and somatic pain from the incision site are the primary causes of immediate postoperative pain following PCNL. Incisional pain is transmitted through T-8 to T-12, whereas visceral pain is largely communicated through the T-10 to L-2 spinal neurons [1]. Several postoperative analgesic options such as oral or parenteral opioids, non-steroidal anti-inflammatory drugs (NSAIDs), intrathecal opioids, and local anesthetics have been evaluated to treat postoperative pain following PCNL [2]. The administration of opioids is associated with many adverse effects, such as nausea, respiratory depression, sedation, and pruritus.

Forero and colleagues first described the ultrasound-guided erector spinae plane (ESP) block in 2016 for the management of thoracic neuropathic pain and postoperative pain in thoracic surgery [3]. However, it has recently been used in postoperative pain management in a variety of procedures, from those on the shoulder to those on the hip [4-6]. In the ESP block, a local anesthetic is injected deep into the erector spinae muscle under ultrasound guidance, which leads to muscle separation from the posterior surface of the transverse process. When compared to neuraxial procedures, the ESP block technique has fewer contraindications and a reduced risk profile [5]. Local anesthetic activity at the ventral and dorsal rami of the spinal neurons is the proposed mechanism of action of the ESP block, but this is currently under investigation and not fully understood [7-10]. As the ultrasound-guided ESP block is a newer technique for postoperative analgesia in PCNL surgery, we conducted this study to determine its practical utility in postoperative pain management with the primary objective of comparing the postoperative pain score. The secondary objectives of the study were a comparison of the duration of analgesia, the total analgesic requirement in 24 hours, and patient satisfaction.

## Materials and methods

This double-blind, randomized controlled study was conducted after receiving approval from the institutional ethics committee (EC number: 156/2020-21 dated 21/06/2021) and the scientific committee of our tertiary care medical college. The study was registered prospectively with the Clinical Trial Registry of India (www.ctri.nic.in), with the registration number CTRI/2022/10/046348. All procedures were conducted according to the Declaration of Helsinki over one year. A total of 60 patients who were posted for elective PCNL surgery, with class I or II physical status according to the American Society of Anesthesiologists, between 35 and 65 years of age, and with a body mass index of 18-30 kg/m^2^ were included in the study. The trial did not include patients with neurological deficits; cardiopulmonary, hepatorenal, or metabolic diseases; anticoagulants; any drug allergies; those with local infection at the proposed site; or pregnant females.

In our study, the difference in Visual Analog Scale (VAS) scores within the first hour after surgery between two groups was considered significant when it was at least 2 points apart. We calculated this variability using a preliminary analysis, with a standard deviation of 1.7. We calculated the sample size using G Power for Windows (Dusseldorf, Germany). The minimum estimated sample size was 24 in each group considering an α value of 0.05, and the power of the study was 0.85. We decided to include 30 patients in each group considering the possibility of dropout or any failure of the block. We used the sealed-envelope randomization method to divide patients into two groups. The anesthesiologist who performed the block was not involved in data collection, and the observer who took all the pain scores was also unaware of the group allocation.

After obtaining informed written consent, the patients fasted for eight hours and received 0.5 mg of alprazolam (oral) on the night before surgery. An intravenous (IV) cannula was inserted in the operation theater. Monitoring of vital parameters such as oxygen saturation, heart rate, non-invasive blood pressure, and electrocardiogram was done continuously. Patients were premedicated with IV 5 µg/kg glycopyrrolate, 0.05 mg/kg midazolam, and 2 µg/kg fentanyl. Induction was achieved by 2 mg/kg propofol, and intubation was facilitated with 0.08 mg/kg vecuronium. Anesthesia was maintained with 50% of nitrous oxide in oxygen and isoflurane along with 0.02 mg/kg vecuronium for maintenance dosing as required. All hemodynamic parameters were monitored continuously and recorded at 30-minute intervals.

**Figure 1:**
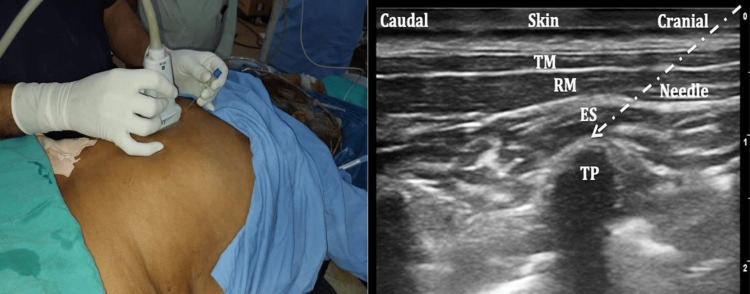
Left: Position of the ultrasound probe in the parasagittal plane. Right: Ultrasound image showing needle position below the erector spinae muscle. TP = transverse process; ES = erector spinae muscle; RM = rhomboid major muscle; TM = trapezius muscle

After the completion of the surgery, an ESP block was applied in the prone position on the surgical side in group E. To visualize the back muscles, trapezius above, rhomboid major in the middle, and erector spinae muscle below, we positioned a high-frequency Sonosite M-Turbo (HFL 38x/6-13 MHz) linear ultrasound transducer in a sterile sheath 3-cm lateral to the T-9 spinous process after proper asepsis (Figure 1). Using an in-plane superior-to-inferior approach, a 23G 10-cm block needle was introduced until it contacted the transverse process (TP) crossing all muscles, and injected 1 mL of the local anesthetic (LA) mixture to delineate the plane. Once hydrodissection of the interfacial plane between the erector spinae muscle and the TP was confirmed, the remaining 19 mL of the LA mixture was injected, which included 10 mL of 0.5% bupivacaine, 5 mL of 2% lignocaine, 1 mL (50 µg) of fentanyl, and 4 mL of normal saline (NS). In group C, the patients received an injection of 20 mL of normal saline using a similar technique.

All anesthetics were discontinued after the injection of the study drug, and once spontaneous breathing started, the residual neuromuscular blockade was reversed using 0.05 mg/kg neostigmine and 10 µg/kg glycopyrrolate, and extubation was performed after proper suctioning. All patients received IV paracetamol 15 mg/kg as an analgesic before being transferred to the postoperative ward. The pain was assessed at 30 minutes and two, four, six, eight, twelve, and twenty-four hours following the block using VAS scores, where 0 represented no pain and 10 represented the worst pain. When necessary or whenever the VAS score in either group was above 4, 100 mg of IV tramadol (400 mg maximum) was administered as a rescue analgesic in the postoperative period. The amount of total analgesic required in both groups during the first 24 hours after surgery was recorded. The patient’s satisfaction was assessed immediately after and 12 hours after surgery in the postoperative care unit using a seven-point Likert verbal rating scale (i.e., 1 = extremely dissatisfied, 2 = dissatisfied, 3 = somewhat dissatisfied, 4 = undecided, 5 = somewhat satisfied, 6 = satisfied, 7 = extremely satisfied) [6]. All patients were monitored for hemodynamic parameters and side effects for up to 24 hours.

Statistical analysis

The statistical analysis of the data was performed using SPSS version 20 (IBM Corp., Armonk, NY, USA). An unpaired t-test was used to analyze the quantitative data, which were reported as means ± standard deviations. The chi-square test was used to analyze the qualitative data presented as numbers and percentages. P-values <0.05 were considered statistically significant, and p-values <0.001 were considered highly significant.

## Results

**Figure 2:**
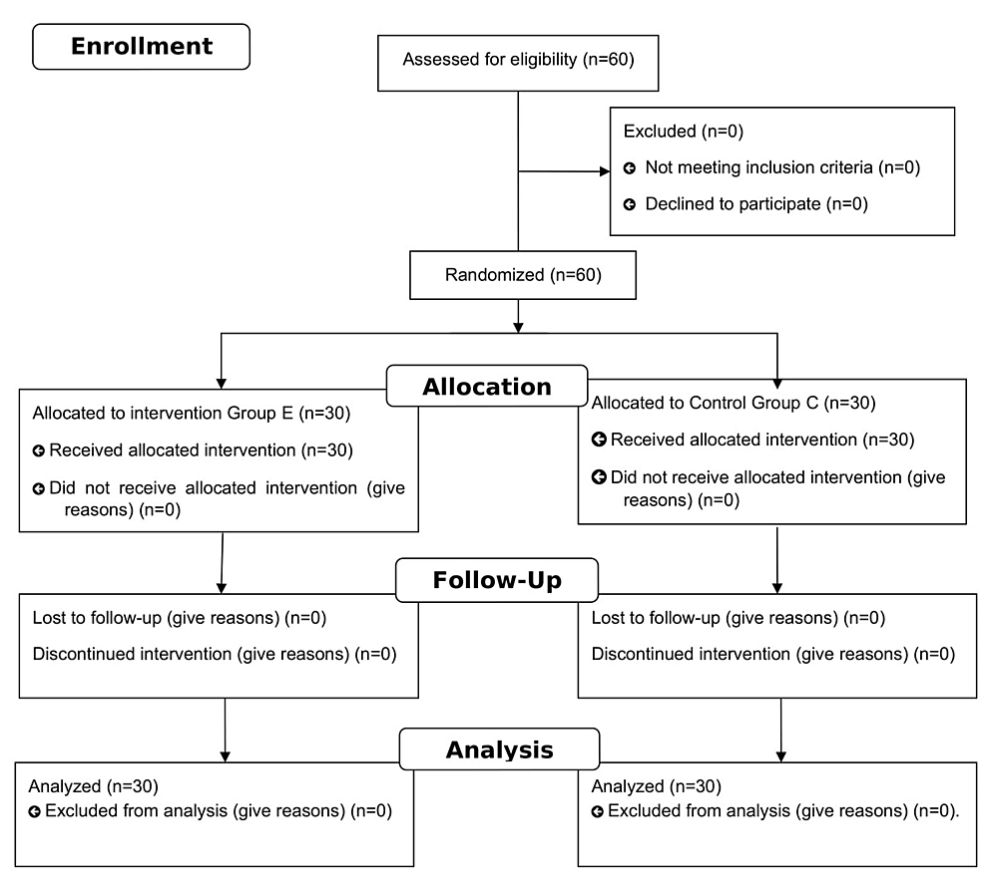
Consort flow diagram. Group E = erector spinae plane block group; group C = control group

All enrolled participants completed the study, as seen in Figure 2.

Regarding patient demographic profiles and clinical characteristics, there were no significant differences (p > 0.05) between the groups. The age, height, weight, and length of the procedure were comparable between both groups, as seen in Table 1.

**Table 1:** Demographic profile of patients and duration of surgery. BMI = body mass index; SD = standard deviation; Group E = erector spinae plane block group; Group C = control group

	Group E		Group C		P-value
	Mean	±SD	Mean	±SD	
Age (years)	46.53	±8.57	44.33	±8.82	0.166
Height (m)	1.64	±0.05	1.64	±0.05	0.289
Weight (kg)	59.40	±5.55	60.40	±5.08	0.235
BMI (kg/m^2^)	22.20	±1.68	22.40	±1.82	0.332
Duration of surgery (minutes)	100.00	±18.19	106.00	±15.22	0.086

**Figure 3:**
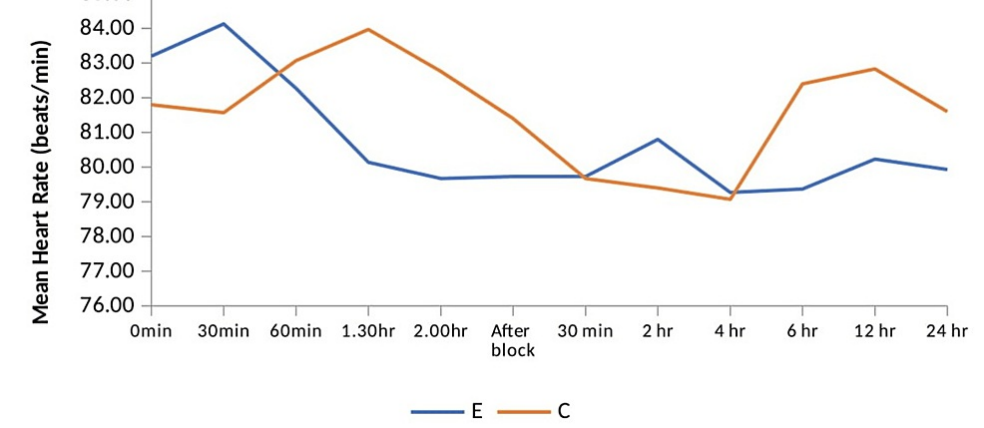
Comparison of mean heart rate (beats/minute) between two groups. Group E = erector spinae plane block group; group C = control group

**Figure 4:**
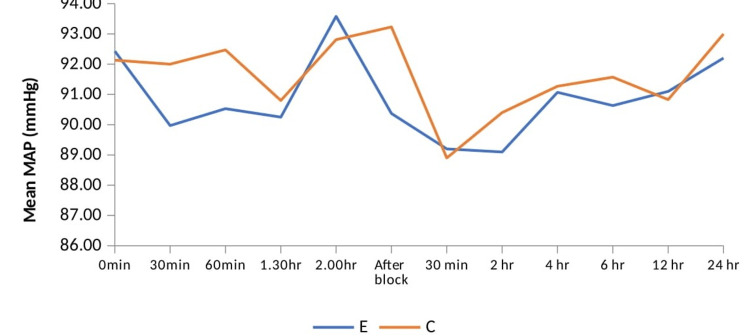
Comparison of mean arterial pressure between two groups across the time periods. Group E = erector spinae plane block group; group C = control group; MAP = mean arterial pressure

Heart rate and blood pressure were comparable between both groups during the intraoperative and postoperative periods, as seen in Figure 3 and Figure 4, respectively.

**Figure 5:**
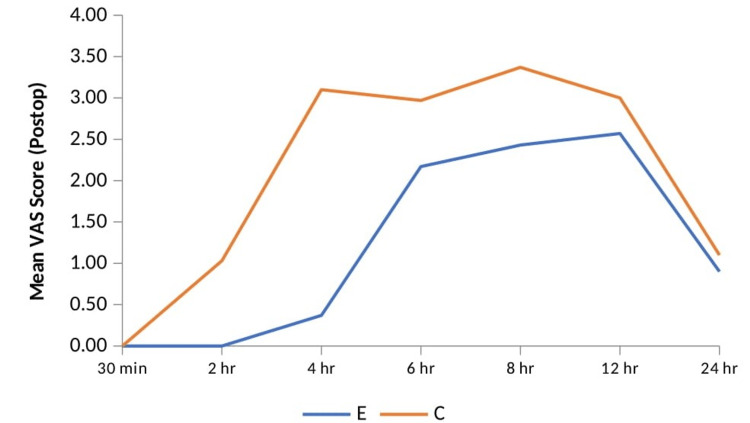
Comparison of the mean VAS score between the two groups. Group E = erector spinae plane block group; Group C = control group; VAS = Visual Analog Scale

At all time intervals, the VAS scores in group E were lower than those in group C. When comparing group E to group C, the difference in VAS scores was highly statistically significant (p < 0.001) at two hours and four hours postoperatively and significant (p < 0.05) at six hours and eight hours postoperatively. Compared with group E, the patients in group C experienced earlier onset of pain, as seen in Table 2 and Figure 5.

**Table 2:** Comparison of VAS score between two groups. * = significant; ** = highly significant; VAS = Visual Analog Scale; Group E = erector spinae plane block group; Group C = control group

VAS score (postoperative)	Group E		Group C		P-value (E vs. C)
	Mean	±SD	Mean	±SD	
30 minutes	0.00	±0	0.00	±0	
2 hours	0.00	±0	1.03	±0.96	0.001^**^
4 hours	0.37	±0.56	3.10	±1.58	0.001^**^
6 hours	2.17	±1.26	2.97	±1.71	0.022^*^
8 hours	2.43	±1.45	3.37	±1.79	0.015^*^
12 hours	2.57	±1.38	3.00	±1.11	0.093
24 hours	0.90	±0.71	1.10	±0.76	0.148

**Figure 6:**
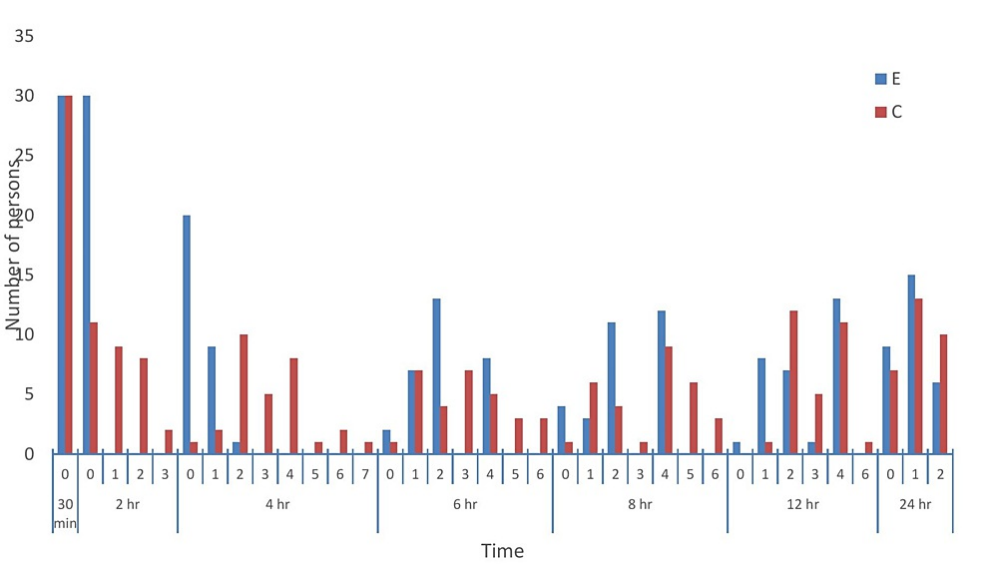
Bar diagram showing the pattern of the VAS score in both groups with respect to time. Group E = erector spinae plane block group; group C = control group; VAS = Visual Analog Scale

Figure 6 shows a bar diagram depicting the pattern of VAS in two groups. The x-axis shows the number of patients in both groups, and the y-axis shows the VAS score at various times. The data are shown in Table 3.

**Table 3:** Comparison of the number of patients experiencing pain as depicted by the VAS score at a particular time. VAS = Visual Analog Scale; Group E = erector spinae plane block group; Group C = control group

Time	VAS score	Group E	Group C
30 minutes	0	30	30
2 hours	0	30	11
	1	0	9
	2	0	8
	3	0	2
4 hours	0	20	1
	1	9	2
	2	1	10
	3	0	5
	4	0	8
	5	0	1
	6	0	2
	7	0	1
6 hours	0	2	1
	1	7	7
	2	13	4
	3	0	7
	4	8	5
	5	0	3
	6	0	3
8 hours	0	4	1
	1	3	6
	2	11	4
	3	0	1
	4	12	9
	5	0	6
	6	0	3
12 hours	0	1	0
	1	8	1
	2	7	12
	3	1	5
	4	13	11
	6	0	1
24 hours	0	9	7
	1	15	13
	2	6	10

The first demand for analgesics was later in group E at 8.87 ± 2.45 hours compared to 5.67 ± 1.58 hours in group C, and the difference was highly statistically significant (p < 0.001). Tramadol was consumed cumulatively less in group E with a mean value of 133.33 ± 47.95 mg compared to 286.67 ± 62.88 mg in group C. Thus, total analgesic consumption over 24 hours was significantly less in group E compared to group C (p < 0.001), as seen in Table 4.

**Table 4:** Comparison of analgesic requirement between the two groups. * = significant; ** = highly significant; SD = standard deviation; Group E = erector spinae plane block group; Group C = control group

Number of analgesic demand in 24 hours	Group E		Group C		P-value
	N	%	N	%	
Demand 1	20	66.67%	0	0.00%	0.001^**^
Demand 2	10	33.33%	8	26.67%	0.287
Demand 3	0	0.00%	18	60.00%	0.001^**^
Demand 4	0	0.00%	4	13.33%	0.019*
	Mean	±SD	Mean	±SD	
Time of first analgesic demand (hours)	8.87	±2.45	5.67	±1.58	0.001^**^
Total analgesic consumption in 24 hours (mg)	133.33	±47.95	286.67	±62.88	0.001^**^

There was a highly statistically significant (p < 0.001) difference in patient satisfaction score at 12 hours postoperatively, with a mean value of 6.73 ± 0.45 in group E compared to 5.87 ± 0.35 in group C. However, no significant difference was observed in patient satisfaction scores immediately after surgery between the two study groups, as seen in Table 5.

**Table 5:** Comparison of patient satisfaction score between the two groups. SD = standard deviation; ** = highly significant; Group E = erector spinae plane block group; Group C = control group

Patient satisfaction score	Group E		Group C		P-value (E vs. C)
	Mean	±SD	Mean	±SD	
Immediately after surgery	5.97	±0.18	5.90	±0.31	0.154
12 hours after surgery	6.73	±0.45	5.87	±0.35	0.001^**^

None of the participants experienced nausea or vomiting, and there was no other drug-related side effect. In the postoperative period, none of the patients experienced any additional serious adverse effects such as bradycardia, hypertension, respiratory depression, or other drug-related reactions that required treatment.

## Discussion

Following PCNL surgery, postoperative pain lowers physical function, slows recovery, increases opioid use, increases morbidity, and raises healthcare costs. Effective pain management promotes recovery and early ambulation. It is mandatory to lower the postoperative stress response after surgery and make rehabilitation easier. In various abdominal surgeries, a multimodal regimen for postoperative analgesia is established, but ESP block is a favorable alternative that is being used recently.

In the present study, VAS scores were lower in group E compared to group C. The difference was highly significant at two and four hours postoperatively, and at six and eight hours, the difference was significant. In a study by Lomate et al., VAS scores in the ESP group were considerably lower at eight and twelve hours [11]. Similarly, Prasad et al. found that VAS scores were considerably lower in the ESP group at all time intervals following PCNL surgery [12]. It was also consistent with our results.

In our study, the first demand for analgesics was earlier in group C than in group E. In a study by Lomate et al., the ESP group had a longer period before their first demand for analgesics than the peritubular infiltration block group (p < 0.001) [11]. Similarly, Srinivasan et al. examined the time before the first demand for analgesics in ESP and local infiltration groups in patients undergoing PCNL surgery and found a significantly (p < 0.001) prolonged duration of analgesia in the ESP group [13].

Gultekin et al. conducted a similar study in which they examined the time to first demand of analgesic in patients after PCNL surgery in ESP and conventional analgesia groups [14]. The time to first demand of analgesics was found to be significantly greater in the ESP group than in the conventional group (172.33 ± 180.5 minutes vs. 84.33 ± 71.12 minutes), which was consistent with the results of our study.

In our study, the total analgesic demand for postoperative analgesia was less in group E than in group C. Similar to our study, Lomate et al. found that the ultrasound-guided ESP block group consumed fewer analgesics overall in the 24-hour postoperative period (148.33 ± 24.51 mg vs. 51.92 ± 45.78 mg) [11]. Similarly, Srinivasan et al. [13] found that the ESP group consumed much less tramadol in the first 24 hours following surgery than the local infiltration group. In agreement with our investigation, Prasad et al. found that the overall dose of rescue analgesia was significantly decreased in the ESP group [12].

At 12 hours postoperatively, there was a highly significant difference in patient satisfaction scores between the groups in our study. However, there was no discernible difference between the two groups in the immediate postoperative period. According to Aydin et al., there were statistically more satisfied patients in the ESP group than in the NSAID group (p < 0.001) [15]. Similarly, Shen Qi-Hong et al. also observed that patient satisfaction was greater in the ESP group (p < 0.01) [16]. Furthermore, Ibrahim et al. [17] used the Likert verbal rating scale to measure patient satisfaction and found that the block group had a greater number of satisfied patients (p = 0.02) than the control group.

Study limitations

The short follow-up period of our study made it impossible to evaluate long-term impacts, which is one of its shortcomings. Research with a larger sample size will be needed to detail the same for other abdominal and thoracic operations. Further studies are recommended to determine the ideal dose and volume of local anesthetic in the ESP block.

## Conclusions

ESP block showed a significant reduction in postoperative pain scores and total analgesic consumption. The duration of analgesia was prolonged with higher patient satisfaction. It demonstrated overall good clinical performance. Thus, it can be said that postoperative analgesia for PCNL surgery using an ultrasound-guided ESP block was proven beneficial.
